# Determination of donepezil in spiked rabbit plasma by high-performance liquid chromatography with fluorescence detection

**DOI:** 10.1098/rsos.181476

**Published:** 2019-01-16

**Authors:** Fardous A. Mohamed, Pakinaz Y. Khashaba, Reem Y. Shahin, Mohamed M. El-Wekil

**Affiliations:** 1 Department of Pharmaceutical Analytical Chemistry, Faculty of Pharmacy, Assiut University, Assiut, Egypt; 2 Drug Research Center, Assiut University, Assiut, Egypt; 3 Department of Pharmaceutical Analytical Chemistry, Faculty of Pharmacy, Deraya University, El-Minya, Egypt

**Keywords:** donepezil, RP-HPLC-fluorescence detection, silodosin, dispersive liquid–liquid microextraction, stability studies

## Abstract

The aim of this paper is to develop sensitive, accurate, reproducible and robust RP-HPLC with fluorescence detection for estimation of donepezil (DZ) in rabbit plasma using silodosin as the internal standard (IS). The prepared samples were quantified on reversed phase column Luna C_18(2)_ (150 × 4.6 mm i.d., 5 µm particle size) operated at room temperature using the mobile phase consisting of methanol: 0.1% acetic acid (50 : 50, v/v) at a flow rate of 1 ml min^−1^. The method was fully validated according to bioanalytical validation guidelines of FDA in terms of system suitability, selectivity, sensitivity, precision and stability. It was found that the increase in peak areas followed the increase of DZ concentration in the range of 2.56–200.00 ng ml^−1^ with LOD of 0.85 ng ml^−1^. The method was successfully applied for the determination of DZ in rabbit plasma using manual shaking dispersive liquid–liquid microextraction.

## 1. Introduction

**Figure 1.**
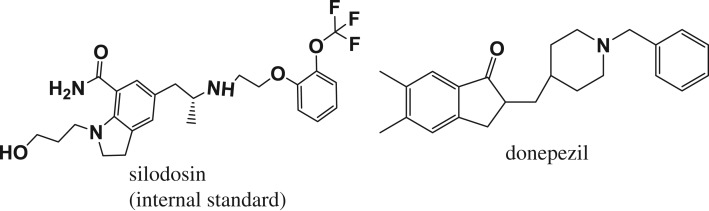
The chemical structures of donepezil and silodosin.

Donepezil (DZ) (figure 1) is a centrally acting reversible acetylcholinesterase inhibitor. It acts by blocking the action of acetylcholinesterase enzyme responsible for the destruction of acetylcholine [1]. The decrease in the levels of acetylcholine in the brain is thought to be responsible for some of Alzheimer's disease symptoms and by blocking the action of acetylcholinesterase enzyme the levels of acetylcholine in the brain will be increased. This may lead to improvement in the memory and cognition functions [1]. About 50% of acetylcholinesterase activity inhibition was achieved at DZ plasma concentration of 15.6 ng ml^−1^ and inhibition plateau at 50 ng ml^−1^ [2]. A few methods were developed for the determination of DZ, including spectrophotometry [3,4] and voltammetry [5,6], that suffer from low sensitivity and are not applied for analysis of the cited drug in biological fluids. The HPLC/UV methods [7–9] suffer from low sensitivity and long analysis time. Moreover, CE/UV [10] and LC/MS [11] have several disadvantages, such as expensive instrumentation (for both), reduced sensitivity and decreased resolution for CE [12] if compared to HPLC methods. Fluorescence detection in HPLC is superior to UV detection because it offers more sensitivity and selectivity. As DZ exists in very low concentrations in plasma, our proposed method was based on HPLC with fluorescence detection. To the best of our knowledge, two HPLC methods coupled to fluorescence detection are reported [13,14]. The first method has a major drawback of long analysis time (about 15 min) and the second one has used micellar liquid chromatography to improve the retention and selectivity, but this technique has a major drawback of reduced efficiency of separation due to micelle formation [15,16]. Our aim is to develop a simple, ultrasensitive, cost-effective, time-saving and fully validated RP-HPLC method for quantitation of DZ in rabbit plasma using fluorescence detector and dispersive liquid–liquid microextraction (DLLME) to enhance sensitivity and selectivity towards its analysis in different matrices. DLLME is a common and popular method for extraction of analytes from aqueous phases. It uses the following ternary solvents: water immiscible extraction solvent, water miscible dispersive solvent and aqueous phase containing the measured analyte. Several forms of DLLME were introduced, such as ultrasound-assisted [17], air-assisted [18], surfactant-assisted [19] and microwave-assisted [20]. The manual shaking-assisted dispersive liquid–liquid microextraction (MSDLLME) [21] was recently used in sample preparation prior to the sample analysis to save extraction equipment, reduce organic solvent consumption in addition to simplicity, rapidity and high enrichment factor. Moreover, the proposed method was applied for the analysis of DZ under different conditions of storage.

## 2. Experimental procedure

### 2.1. Chemicals and reagents

DZ and silodosin (figure 1) were obtained as a gift from NODCAR (National Organization of Drug Control and Research), El-Giza, Egypt. Aricept^®^ tablets (Pfizer, Cairo, Egypt) are labelled to contain 10 mg DZ. Methanol and acetonitrile were of HPLC grade (Fischer chemicals, UK), glacial acetic acid (BDH chemicals, UK), double distilled water was purified using a Milli-Q plus cartridge purification system (Millipore, Waters, USA) to get ultra-pure water of 18 MΩ.

### 2.2. Instrumentation and chromatographic conditions

Analysis was carried out on Thermo^®^ HPLC line system (UK) equipped with a binary pump (HPG-3200 SD) operated in an isocratic mode at a flow rate of 1 ml min^−1^, an auto sampler (WPS-3000 TSL), fluorescence multi-wavelength detector (FLD-3400 RS detector). Chromatographic separation was performed on Luna C_18(2)_ (150 × 4.6 mm i.d., 5 µm particle size; Phenomenex, USA) protected by a pre-column (guard column with C_18_ precolumn inserts; Phenomenex, USA). The HPLC system control and data processing were performed by software Chromeleon 7^®^. Mobile phase consisted of methanol: 0.1% acetic acid (50 : 50, v/v) and the elution was carried out in an isocratic mode. The mobile phase was filtered through nylon filtration membrane (0.45 mm in diameter, 0.2 µm pore size) using vacuum filtration unit (Phenomenex, USA). The injection volume was 20 µl. Determination was performed at *λ*_ex_ 269 nm and *λ*_em_ 390 nm using silodosin as the internal standard (IS).

### 2.3. Preparation of standard solutions

An accurately weighed amount of 10 mg of DZ was transferred into a 100 ml volumetric flask, dissolved in 50 ml methanol and then completed to the mark by the same solvent to obtain a stock standard solution containing 100 µg ml^−1^ of DZ. The same method was used to obtain a stock methanolic solution of 10 µg ml^−1^ silodosin as the IS. These stock standard solutions were stored in the dark at 4°C for one week and were stable under these conditions, as indicated by the constancy of the peak area. Working standard solutions were prepared daily from the stock standard solution by serial dilution with the mobile phase, it was also used for the preparation of spiked plasma samples for the construction of spiked plasma calibration curve.

Three quality control samples (10, 100, 200 ng ml^−1^) covering low-, medium- and high-quality control samples (LQC, MQC, HQC) were prepared, respectively, and stored at 2–8°C until analysis.

### 2.4. Rabbit plasma

#### 2.4.1. Preparation of rabbit plasma

Blood from six healthy rabbits with average weights of 2 kg was placed in the tube treated with heparin anticoagulant. The cells of blood were removed by centrifugation at 3000 r.p.m. for about 10 min. The supernatant was collected, bottled and stored below −10°C.

#### 2.4.2. Plasma sample stability

The stability of DZ in rabbit plasma was assessed by preparing three sets of QC samples at low (10 ng ml^−1^), medium (100 ng ml^−1^) and high (200 ng ml^−1^) concentrations in the same manner as the calibration standards and stored at −80°C until use. Another QC sample was freshly prepared and analysed immediately at zero time to evaluate the accuracy and precision of this method.

The stability study of plasma samples included bench top stability study where triplicate of low, medium and high QC samples was allowed to stand on the bench for 48 hr at room temperature (25°C ± 2). Long-term stability study was done by storing triplicate of low, medium and high QC samples at –80°C for eight weeks before being analysed, while the freeze–thaw stability study was determined at low, medium and high QC concentration after freezing (–80°C) for 24 h and thawing completely at room temperature (25°C ± 2) for three cycles.

#### 2.4.3. Stock solution stability

The stock solution stability was evaluated by appropriate dilution of the stock solution with the mobile phase to obtain low, medium and high concentrations (at 10, 100, 200 ng ml^−1^, respectively) and analysed after storage for 48 h at room temperature (25°C ± 2) and after storage at −80°C for eight weeks.

### 2.5. Practical application

#### 2.5.1. Sample preparation

Ten tablets were accurately weighed and finely powdered. Portion equivalent to one tablet was accurately weighed and dissolved in 50 ml methanol and the pH was adjusted to 10.0 (by 0.1 N NaOH to liberate DZ base from its hydrochloride salt). The contents were sonicated for 30 min to ensure complete solubility of liberated DZ base. The filtrate was transferred to the 100 ml volumetric flask and diluted to the mark with methanol. Working solutions were prepared by further dilution of the filtrate with the mobile phase.

#### 2.5.2. Preparation of rabbit plasma

##### 2.5.2.1. Manual shaking-assisted dispersive liquid–liquid extraction procedure for rabbit plasma

**Scheme 1.**
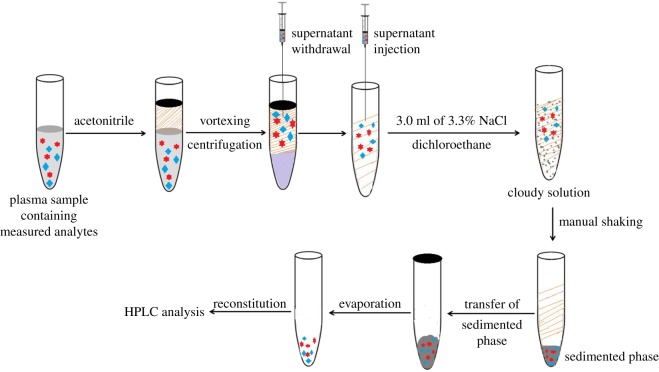
Representative diagram for preparation of rabbit plasma containing measured analytes by MSDLLME.

Rabbit plasma (100 µl) was spiked with various amounts of DZ and 20 µl of IS (200 ng ml^−1^). A total of 600 µl of acetonitrile as protein precipitant and dispersive agent was used. It was then vortexed at high speed for 1 min and centrifuged at 5000 r.p.m. for 30 min. The supernatant was withdrawn and transferred into another Eppendorf tube. After that, 3 ml of 3.3% NaCl (w/v) (adjusted to pH 9.0) and 150 µl of dichloroethane (as the extracting solvent) were added. A cloudy solution was formed before shaking for 20 s (20 repetitions). All extracting phase was transferred to a small tube and evaporated under a stream of nitrogen. The residue was reconstituted in 100 µl of the mobile phase and then the steps of general procedure were applied. Schematic presentation of the proposed MSDLLME for extraction of DZ from plasma is shown in scheme 1.

## 3. Results and discussion

### 3.1. Optimization of chromatographic conditions

**Figure 2.**
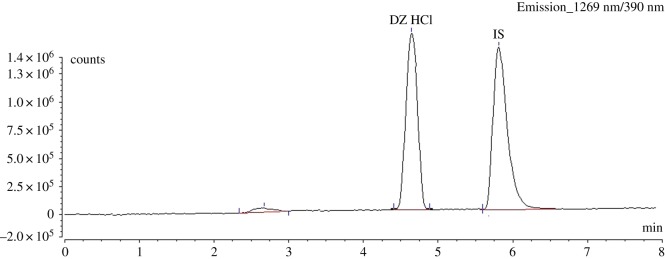
Chromatogram for the determination of DZ (100 ng ml^−1^) using silodosin as IS (200 ng ml^−1^), the first peak is the solvent front.

Well-defined symmetrical peaks were obtained upon measuring the response of the eluent under the performance parameters, as shown in figure 2. Optimum conditions affecting the chromatographic performance are obtained after thorough experimental trials that could be summarized as follows.

#### 3.1.1. Effect of organic modifier type and ratio

The effect of the organic modifier type on the development and elution of peaks was investigated by using acetonitrile and methanol. The good peak symmetry, column performance and efficiency were obtained using methanol. Therefore, methanol was selected as an ideal organic solvent for DZ. The effect of methanol ratio on the chromatographic behaviour of the eluted peak was studied (electronic supplementary material, figure S1). It was observed that a mobile phase system consisting of 50% methanol gave acceptable *R_t_* (about 4.9 min) which is better expressed as the capacity factor (*K*^′^) value.

#### 3.1.2. Effect of aqueous phase type and its concentration

Different aqueous phases were investigated including phosphate, acetate and citrate buffers (20 mM) and 0.1% acetic acid (pH 3.5). The study proved that using 0.1% acetic acid showed the best sensitivity and peak symmetry (table 1). Moreover, different concentrations of acetic acid were investigated in the range of 0.04–0.15%, *K*^′^ and peak symmetry were monitored each time. It was found that *K*^′^ was not affected, but unacceptable peak symmetry was obtained in the range of 0.04–0.08% acetic acid and reasonable peak symmetry with good sensitivity was observed in the range of 0.08–0.15% acetic acid. Therefore, 0.1% acetic acid (pH 3.5) was selected as the optimum concentration for the determination of DZ.

**Table 1.** The effect of changing aqueous phase in the mobile phase on the efficiency and performance of the Luna C_18(2)_ column.

type of buffer	*N*^a^	*A_s_*^b^
phosphate	7853	1.3
acetate	8461	1.2
citrate	7294	1.4
0.1% acetic acid	10 723	1.0

^a^Number of theoretical plates.

^b^Asymmetry factor at 10% of peak height.

#### 3.1.3. Flow rate

The effect of flow rate on the sensitivity, *A_s_* and *K*^′^ of the eluted peak was studied over the range of 0.5–1.5 ml min^−1^. It was found that a flow rate of 0.5 ml min^−1^ gave an asymmetric delayed peak with reduced sensitivity. At flow rates of 1.2–1.5 ml min^−1^, an overlapping peak with the solvent front was observed. Therefore, the flow rate of 1 ml min^−1^ was optimum for a good separation and resolution at reasonable time.

#### 3.1.4. Internal standard selection

The IS should be completely separated from DZ and with *K*^′^ that keeps the method having reasonable time, enhances the precision of the assay. Therefore, six different drugs having native fluorescence were investigated, namely daclatasvir, sumatriptan, amlodipine, felodipine, propranolol and silodosin. It was found that silodosin gave an acceptable peak symmetry (1.2) at *R_t_* close to that of DZ (at 5.9 ± 0.1 min). As a result, silodosin was selected as a suitable standard for DZ. Figure 2 shows the chromatographic separation of DZ from the IS under the experimental conditions of DZ measurement.

### 3.2. Method validation

The developed procedure was fully validated according to FDA bioanalytical guidelines [22] for selectivity, sensitivity, recovery, linearity, precision and accuracy.

#### 3.2.1. Calibration curve

**Figure 3.**
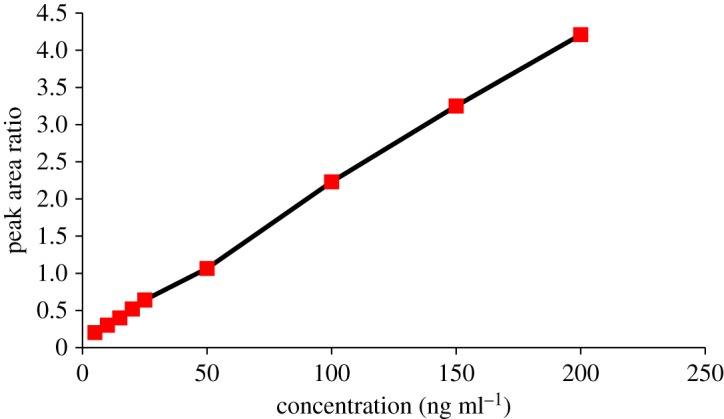
Relationship between peak area ratio (*y*) and concentration (*x*).

The calibration curve of DZ was obtained by plotting the final concentration (*x*) versus the peak area ratios (*y*) of varying concentrations of DZ to a constant concentration of IS (200 ng ml^−1^) and fitted to the regression equation *y* = a + b*x*. Regression analysis was carried out using least-square method [23]. The relationship was found to be linear over the range of 5–200 ng ml^−1^ (figure 3) with small intercepts and good correlation coefficient over the cited concentration range (table 2). The lower limit of quantification (LLOQ) was determined as the lowest concentration with accuracy and precision under acceptable limits by observing for the peak with identifiable, discrete and reproducible nature.

**Table 2.** Quantitative parameters of the proposed RP-HPLC method for the determination of DZ.

parameter	estimated values
linearity range (ng ml^−1^)	5–200
correlation coefficient (*r*)	0.9990
determination coefficient (*r*^2^)	0.9993
intercept (a) ± s.d.^a^	0.0813 ± 0.006
slope (b) ± s.d.^a^	0.0238 ± 0.0004
LOD (ng ml^−1^)	0.85
LLOQ (ng ml^−1^)	2.56

^a^Average of three determinations.

#### 3.2.2. Accuracy

Accuracy of the proposed method was confirmed by the standard addition method, where a known amount of pure standard covering (LLOQ, LQC, MQC and HQC concentrations) was added to the commercially available tablet sample (Aricept^®^ tablets) and the recovery percentage was calculated. Results of % recovery, in table 3, indicate that the proposed method exhibits good accuracy and shows no interference from the excipients.

**Table 3.** Standard addition method for the analysis of DZ by the proposed RP-HPLC method.

pharmaceutical formulation	amount taken (ng ml^−1^)	amount added (ng ml^−1^)	amount found (ng ml^−1^)	% recovery ± s.d.^a^
Aricept^®^ tablets	100	2.56 (LLOQ)	2.44	95.8 ± 2.01
		25 (LQC)	24.87	99.5 ± 1.62
		50 (MQC)	49.96	99.9 ± 0.93
		100 (HQC)	99.89	99.9 ± 1.28

^a^Average of six determinations.

#### 3.2.3. Precision

The precision of an analytical method is the degree of agreement among individual test results and it is usually expressed as the relative standard deviation percentage (% RSD) of a series of measurements. Precision was determined by the measurement of six replicates of four different freshly prepared QC solutions covering LLOQ, low, medium and high concentrations (LQC, MQC, HQC) of DZ and each contained 200 ng ml^−1^ of IS. Both repeatability (intra-day precision) and reproducibility (inter-day precision) were studied. For the evaluation of precision, the % RSD of each concentration is expected to be within ± 15% from the nominal value, except for the LLOQ where it should not be more than 20% of the nominal value. Results in table 4 depict good precision of the proposed HPLC method (% RSD ≤ ranged between 0.26 and 1.15% for the quality control samples), while for the LLOQ it ranged between 0.92 and 1.56%).

**Table 4.** Repeatability and reproducibility for the analysis of DZ by the proposed RP-HPLC method.

concentration (ng ml^−1^)	repeatability		reproducibility	
	% recovery ± s.d.^a^	% RSD	% recovery ± s.d.^a^	% RSD
2.56(LLOQ)	95.83 ± 0.89	0.92	94.2 ± 1.47	1.56
25 (LQC)	97.9 ± 0.26	0.26	98.2 ± 0.39	0.40
100 (MQC)	99.2 ± 0.59	0.59	98.8 ± 0.87	0.88
150 (HQC)	99.7 ± 0.84	0.84	99.3 ± 1.15	1.15

^a^Average of six replicates.

#### 3.2.4. Selectivity

The selectivity of the assay was checked by analysing six Aricept^®^ tablets. Chromatograms were compared with the chromatograms obtained by analysing the standard solution containing DZ. As retention time value is the characteristic for any given compound provided that the same stationary and mobile phases are used, it can provide corroborative evidence to the identity of a compound. In this study, retention times for authentic drug were in pure form 4.9 ± 0.1 and 6.0 ± 0.2 while in tablets were 4.9 ± 0.2 and 6.1 ± 0.2 for DZ and silodosin, respectively. There is no significant difference in the retention time values at different positions between the compared peaks.

#### 3.2.5. Applications of the proposed RP-HPLC method

##### 3.2.5.1. Application of the proposed HPLC method for the analysis of DZ in its tablets

The proposed method was applied successfully for the determination of the studied drugs in the pharmaceutical dosage form (Aricept^®^ 10 mg tablets). Three replicate measurements were made. The results obtained for the analysis of tablets were validated by the comparison with a previously reported method [6]. No significant difference was found by applying *t* and *F* tests at 95% confidence level indicating good accuracy, precision and suitability of the proposed method for determination of the investigated drug in pharmaceutical dosage forms, as shown in table 5.

**Table 5.** Application of the proposed HPLC method for the determination of DZ in its tablet dosage form.

Aricept^®^ tablets (10 mg)		
	proposed method	reported method [6]
recovery ± s.d.^a^	98.1 ± 0.02	97.9 ± 0.02
Student's *t*-test^b^	1.6	2.2
variance ratio *F*-test^b^	2.8	3.4

^a^Average of five determinations.

^b^Theoretical value for *t* and *F* at 95% confidence limit, *t* = 3.36 and *F* = 6.38.

##### 3.2.5.2. Application of the proposed RP-HPLC method for the determination of DZ in rabbit plasma using MSDLLME

###### Extraction recovery

To obtain good optimized conditions, extraction recovery (ER) was used to evaluate extraction conditions. ER is the percentage of total analyte (*P*°) extracted into the extracting phase (*P*_ex_) and can be calculated using the following formula:

3.1 $$\text{\%}\mathrm{ER}=\frac{P_{\mathrm{ex}}}{P^{\circ }}=\frac{C_{\mathrm{ex}}}{C^{\circ }}\;\times \frac{V_{\mathrm{ex}}}{V^{\circ }}\times 100,$$

where *C*_ex_ and *C*° are the concentrations of analyte in supernatant and initial concentration of analyte in the aqueous phase, respectively. The value of *C*_ex_ can be calculated from the calibration curve after injection of standard. *V*_ex_ and *V*° are the volumes of extracting and aqueous phases, respectively. Preconcentration factor (PF) can be defined as the concentration of analyte in extracting aqueous phases.

3.2 $$\mathrm{PF}=\frac{C_{\mathrm{ex}}}{C^{\circ }}.$$

Equations (3.1) and (3.2) result in the following equation:

$$\text{\%ER}=\text{PF}\times \frac{V_{\mathrm{ex}}}{V^{\circ }}\times 100.$$

*Selection of initial experimental conditions.* Organic solvents, e.g. methanol and acetonitrile (ACN) are widely used as protein precipitants and dispersive solvents. In the preliminary steps, acetonitrile was found as a good protein precipitant and dispersive solvent. Moreover, it has good miscibility with the aqueous phase and with MSDLLME gave an excellent recovery for DZ determination.

*Extracting solvent selection.* The most important step in DLLME is the selection of extracting solvent to give good recovery for DZ analysis. The extracting solvent needs assistance to be dispersed into the sample because the dispersing solvent (ACN) is already mixed into the aqueous phase that contains the analyte. So, manual shaking was used due to ease of application. Many extracting solvents were tested, such as dichloroethane, dichloromethane, chloroform, ether and chlorobenzene (250 µl each). The selection criteria include water-immiscibility, higher density than water and high extracting capability for analyte of interest, and on the basis of these criteria, dichloroethane was selected as a good extracting solvent. After the addition of extracting solvent, the extracted phase was then dried due to its potential effect on the chromatographic behaviour, then reconstituted into 100 µl of the mobile phase; extraction efficiency was determined based on the peak area ratio (electronic supplementary material, figure S2).

*Investigation of other factors.* **Different factors affecting the extraction step were optimized, such as volume of extracting solvent, pH, amount of NaCl (ionic strength) and manual shaking time. It should be noted that the volume of acetonitrile was fixed at 600 µl at 100 µl rabbit plasma to ensure complete protein precipitation. It was found that the optimum conditions were 150 µl of dichloroethane, pH = 9.0, concentration of NaCl = 3.3% w/v and shaking time = 20 s (20 repetitions).

*Volume of extracting solvent.* The volume of extracting solvent has an important role in the extraction efficiency. But, when the extracting solvent volume was increased, the final volume of the separated organic phase will be increased, thus the preconcentration factor will be decreased. Meanwhile, the volume of the extracting solvent could not be much reduced because this would lead to decrease in the extraction efficiency of the analyte. To optimize the volume of the extracting solvent, the extraction efficiency of each volume was calculated (electronic supplementary material, figure S3). The volume of dichloroethane was varied from 50 to 200 µl and it was found that the extraction efficiency was increased up to 150 µl, after which it started to decrease, probably due to dilution of the extracted analyte in the extracting solvent. So, a volume of 150 µl of dichloromethane was used throughout the study.

*Ionic strength of NaCl.* The percentage of the salting out agent has played an important role in changing the polarity of the analyte, thus increasing its migration from the dispersing to the extracting solvent leading to increased recovery indicated by percentage ER. Different percentages of NaCl were added in the range of 3.0–3.6% w/v in 0.1% intervals, it was found that the extraction efficiency increased up to 3.2% w/v NaCl, after that the ratio remained constant, as a result 3.3% w/v was selected as the optimum concentration of NaCl (electronic supplementary material, figure S4).

*Effect of pH of NaCl solution.* As the pH of the solution must be high enough to maintain DZ and IS in the un-ionized form, the optimization was carried out in the pH range of 7–11. Results in electronic supplementary material, figure S5, indicate that ER increased from pH 8; after that it became constant, so pH 9 was selected as the optimum pH of NaCl solution.

*Effect of repetitions number in shaking.* Shaking time was held constant at 20 s and repetitions were varied from 10 to 24. As shown in electronic supplementary material, figure S6, the per cent extraction recoveries were increased when repetitions varied from 10 to 16 repetitions, after that it became constant. As a result, 20 repetitions were used as an optimal number.

#### 3.2.6. Analytical performance of MSDLLME-based RP-HPLC for analysis of donepezil in rabbit plasma

##### 3.2.6.1. Calibration curve

The calibration curve of DZ was obtained by plotting the final concentration (*x*) versus the peak area ratios (*y*) of varying concentrations of DZ (six concentrations in three replicates) to a constant concentration of IS (200 ng ml^−1^) and fitted to the equation *y* = a + b*x*. Regression analysis was carried out using least-square method [24]. The relationship was found to be linear over the range of 6–200 ng ml^−1^ with small intercepts and good correlation coefficient over the cited concentration range. This linear relationship gives evidence that the system is performing properly throughout this concentration range. The LLOQ was found to be 4.95 ng ml^−1^ while LOD was found to be 1.64 ng ml^−1^. Quantitative parameters are shown in table 6.

**Table 6.** Quantitative parameters of the proposed MSDLLME-based RP-HPLC method for the determination of DZ in rabbit plasma.

parameter	estimated values
linearity range (ng ml^−1^)	6–200
correlation coefficient (*r*)	0.9990
determination coefficient (*r*^2^)	0.9984
intercept (a) ± s.d.^a^	0.2238 ± 0.0058
slope (b) ± s.d.^a^	0.0118 ± 0.0004
LOD (ng ml^−1^)	1.64
LLOQ (ng ml^−1^)	4.95

^a^Average of three determinations.

##### 3.2.6.2. Accuracy

The accuracy of the proposed method was determined by investigating the recovery percentages of spiked QC plasma samples with DZ and IS at four concentration levels (4.95, 10, 100, 150 ng ml^−1^) representing LLOQ, low, medium and high QC (three replicates of each concentration). The results in electronic supplementary material, table S1, revealed good accuracy and recovery percentages ranging from 98.3 to 98.8%.

##### 3.2.6.3. Precision

The precision was evaluated by measuring intra-day and inter-day RSDs of repeated measurements. The intra-day values of RSD were calculated based on six replicate runs at four different concentrations of spiked blank plasma samples (4.95, 25, 100, 150 ng ml^−1^) representing LLOQ, LQC, MQC and HQC in a day, while the inter-day values of RSD were evaluated using six replicate runs of the same concentrations over a period of 2 consecutive working days. The results in electronic supplementary material, table S2, show good repeatability and reproducibility of the proposed method where the RSD ≤ 1.13% for the QC samples and ≤ 1.24 for the LLOQ, which makes it adequate for application in the quality control laboratories.

##### 3.2.6.4. Selectivity of the proposed method for the determination of DZ in rabbit plasma

**Figure 4.**
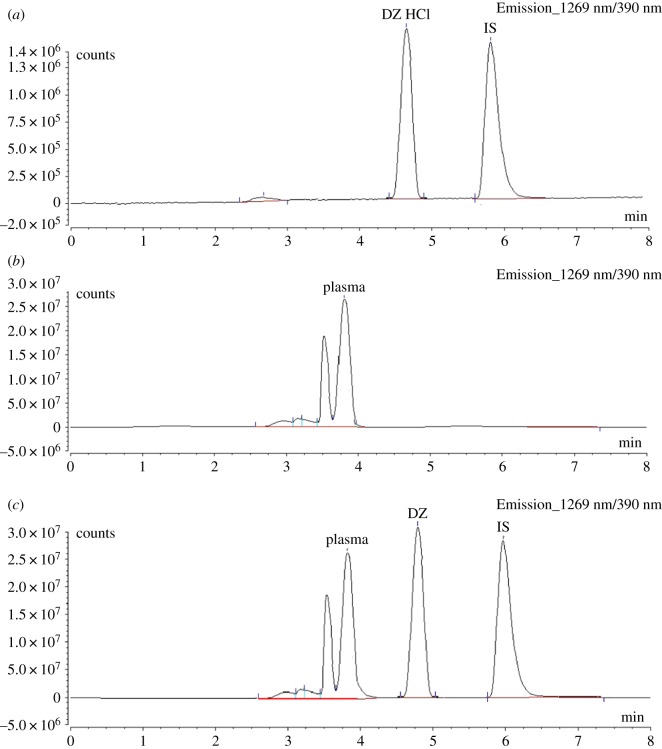
Chromatograms of (*a*) standard solution of 100 ng ml^−1^ of DZ and 200 ng ml^−1^ (IS), (*b*) blank rabbit plasma and (*c*) rabbit plasma spiked with 150 ng ml^−1^ of DZ and 200 ng ml^−1^ (IS) using the proposed RP-HPLC method conditions.

The selectivity of the proposed method was assessed by comparing the chromatograms obtained from the spiked sample with those of the standard and blank plasma (figure 4). Moreover, interference study was carried out by spiking the plasma with several co-administered drugs usually taken with DZ, such as sertraline, olanzapine, risperidone, quetiapine, gabapentin, pregabalin and piracetam (conc. of each was 200 ng ml^−1^). As neither of these co-administered drugs exhibits native fluorescence, there is no interference at the retention time of DZ or the IS.

##### 3.2.6.5. Stability study

Plasma QC sample stabilities at the cited concentrations (10, 100, 200 ng ml^−1^) were used for the stability study. Results in table 7 show that DZ plasma samples are stable in all storage conditions, and, if needed, can be re-analysed after repeated freezing and thawing without compromising the results. It also showed that DZ is stable in its stock solution up to 8 weeks.

**Table 7.** The stability of DZ under various storage conditions.

	recovery % ± s.d.^a^				
	plasma samples			stock solution	
concentration (ng ml^−1^)	short term (48 h)	long term (8 weeks −80°C)	freeze–thaw cycles	short term (48 h)	long term (8 weeks −80°C)
10	98.7 ± 0.26	98.3 ± 0.48	97.6 ± 0.99	99.8 ± 0.63	99.8 ± 0.28
100	99.7 ± 0.85	99.2 ± 0.88	98.7 ± 0.91	99.9 ± 0.48	99.4 ± 0.36
200	99.9 ± 0.74	99.3 ± 0.81	98.9 ± 0.82	98.4 ± 0.19	99.3 ± 0.85

^a^Average of three determinations.

## 4. Comparison to other reported methods

This method was compared to other methods for determination of DZ HCl regarding linearity range, LOD and LLOQ, repeatability (represented by the % RSD) and applicability (table 8). It is noted that UV detection represents a cheap and easily accessible method of analysis, fluorescence detection is of moderate expense and also easily accessible while LC/MS is very expensive and hard to access. From table 8, it is clear that the proposed method offers good sensitivity and hence applicability compared to other methods.

**Table 8.** Comparison of the proposed method for the determination of DZ with reported methods.

detection	linearity range (ng ml^−1^)	LOD (ng ml^−1^)	LLOQ (ng ml^−1^)	precision (% RSD)	applicability	ref.
UV	0.125–16 × 10^3^	0.03 × 10^3^	0.125 × 10^3^	≤1.81	orally disintegrating tablet	[7]
UV	10.0–60.0 × 10^3^	10.0 × 10^3^	—	0.5	tablets	[8]
UV	3.0–90.0	—	3.0	7.3–7.6	human plasma	[9]
fluorescence	5.0–2000.0	—	—	≤ 6.5	tablets, spiked human plasma	[13]
LC/MS	0.15–50.00	—	0.15	≤8.92	human plasma	[11]
fluorescence	5.0–200.0	0.85	2.56	≤ 0.94	tablets, spiked rabbit plasma	this work

## 5. Conclusion

A simple, cost-effective, ultrasensitive, fully validated and time-saving RP-HPLC with fluorescence detection was developed for the estimation of acetyl cholinesterase inhibitor DZ in pure form, tablets and rabbit plasma. The method showed good selectivity, sensitivity, reliability and short analysis time (8 min), compared with previously reported methods. The method was extended for determination of DZ in rabbit plasma after MSDLLME and the obtained results have proved the good applicability of MSDLLME for the analysis of DZ in complexed matrices for further analysis. Thus, the method can be used in quality control laboratories, drug–drug interaction, pharmacokinetic and pharmacodynamic studies of the drug. Moreover, stability studies were performed for evaluation of the different storage conditions on QC plasma spiked with DZ and the obtained results proved the good stability of drug under these stressed conditions.

## Supplementary Material

Supplementary materials

## Data accessibility

All data are included within the article.
