# Supplementary material for: Determination of donepezil in spiked rabbit plasma by high-performance liquid chromatography with fluorescence detection
Source: R Soc Open Sci. 2019 Jan 16;6(1):181476. doi: 10.1098/rsos.181476 (PMC6366186; doi:10.1098/rsos.181476)
Supplement: Supplementary materials [file rsos181476supp1.docx]

**Supplementary materials**

**Determination of donepezil in spiked rabbit plasma by high performance liquid chromatography with fluorescence detection**

Fardous A. Fattah ^a^, Pakinaz Y. Khashaba ^a,b^, Reem Y. Shahin ^c^, Mohamed M. El-Wekil ^a^*

^a^ Department of Pharmaceutical Analytical Chemistry, Faculty of Pharmacy, Assiut University, Egypt.

^b^ Department of Pharmaceutical Analytical Chemistry, Faculty of Pharmacy, Deraya University, El-Minya, Egypt.

^c^ Drug Research Center, Assiut University, Egypt.

* Corresponding author: mohamed.mohamoud@ymail.com

**Figures**

**Figure 1S.** Effect of % MeOH on capacity factor (K^’^).


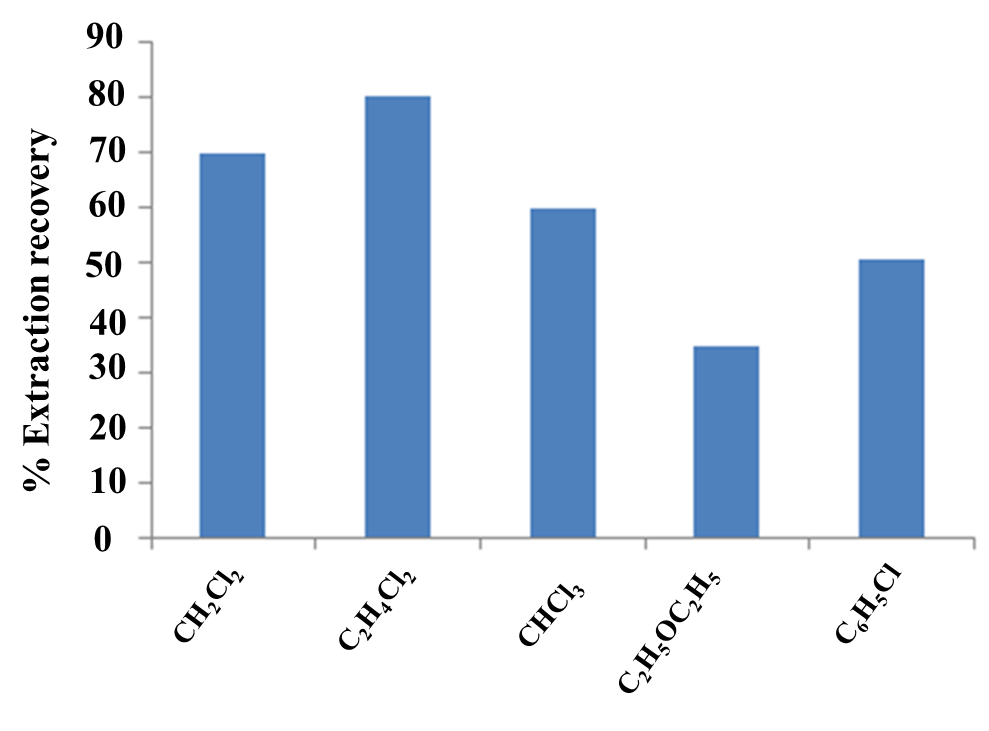


**Figure 2S.** Extraction efficiency according to extraction solvent. DZ concentration is 100 ng mL^-1^, IS concentration is 200 ng mL^-1^.


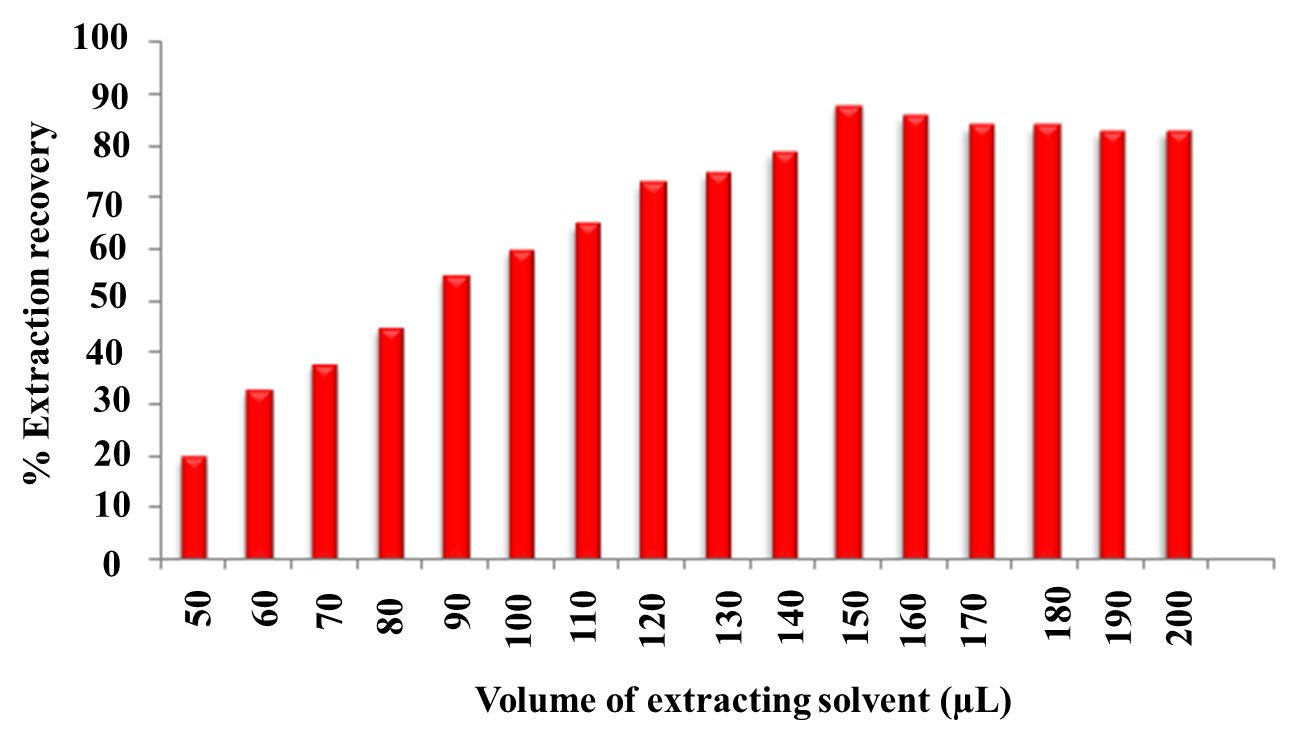


**Figure 3S.** Effect of volume of extraction solvent on the extraction efficiency, DZ concentration, 100 ng mL^-1^, IS concentration 200 ng mL^-1^.


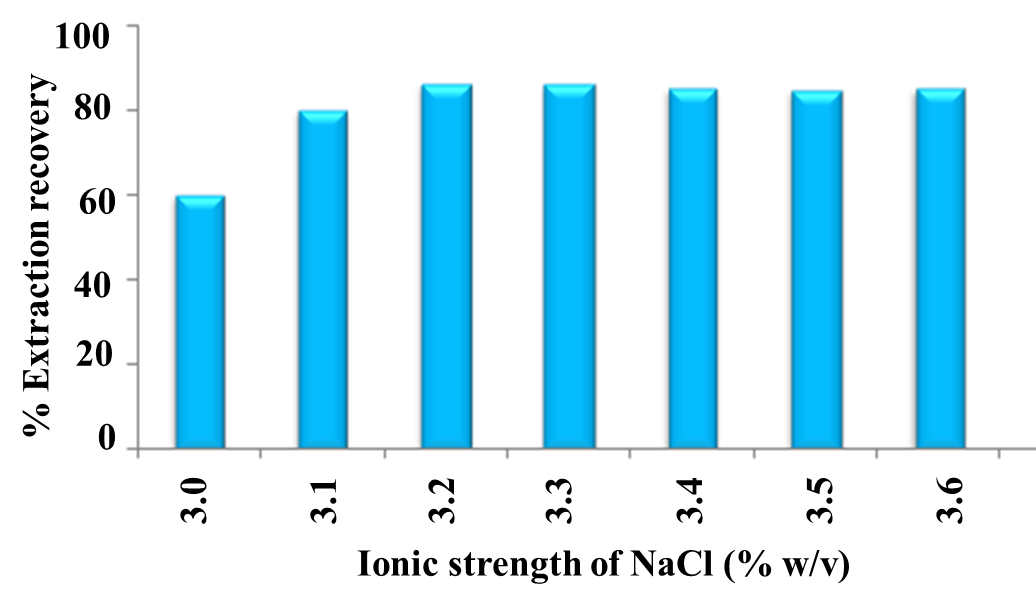


**Figure 4S.** Effect of ionic strength of NaCl (DZ concentration, 100 ng mL^-1^, IS concentration 200 ng mL^-1^).


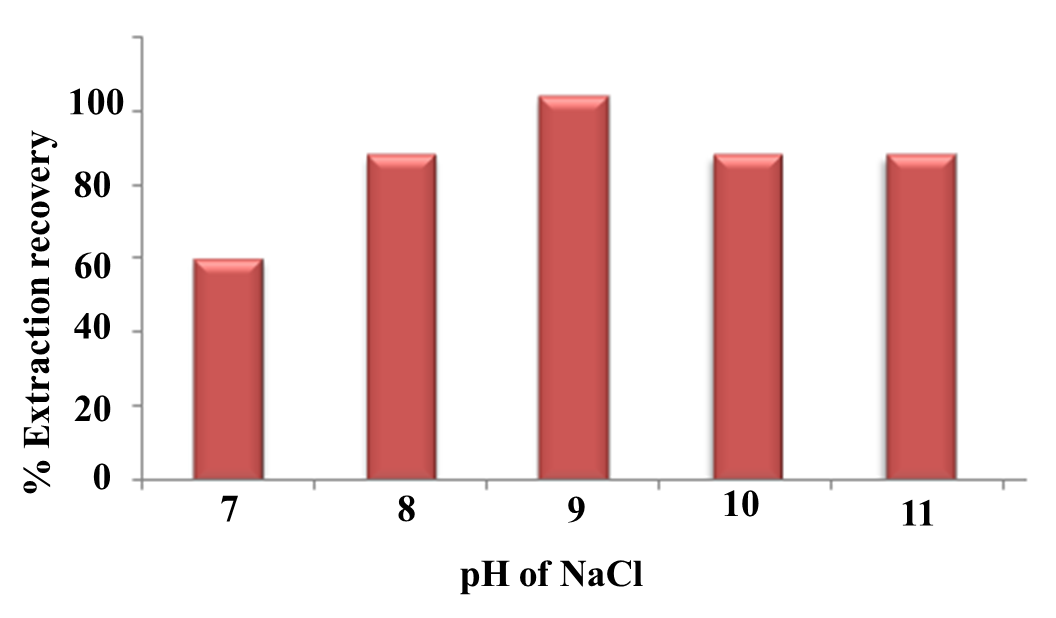


**Figure 5S.** Effect of pH on the salting out capacity of NaCl. (DZ concentration is 100 ng mL^-1^, IS concentration is 200 ng mL^-1^).

**
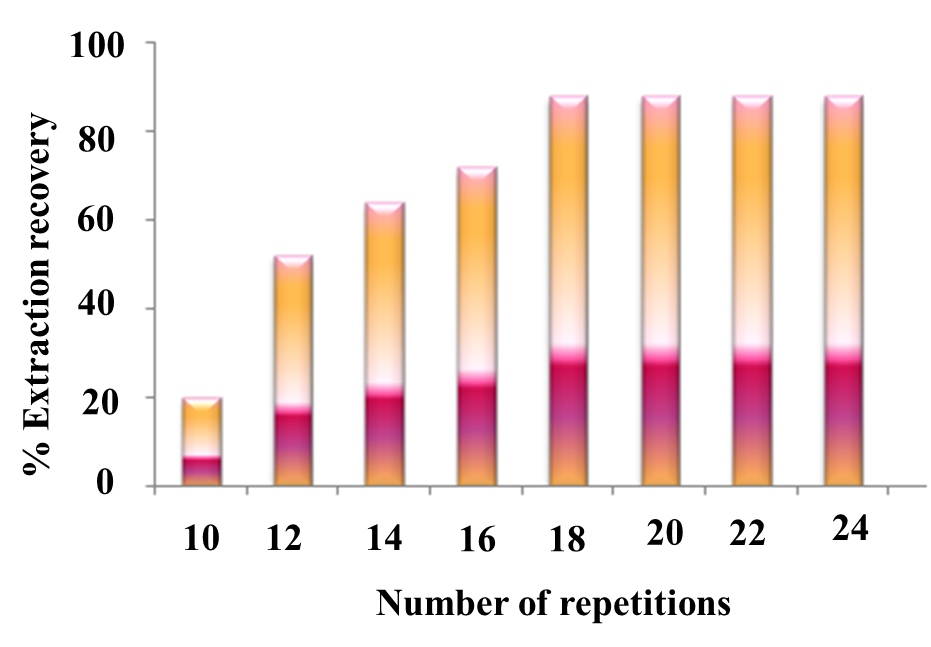
**

**Figure 6S.** Effect of shaking reptitions on the salting out capacity of NaCl. (DZ concentration is 100 ng mL^-1^, IS concentration is 200 ng mL^-1^).

**Tables**

**Table 1S.** Accuracy of the proposed MSDLLME based RP-HPLC method for the determination of DZ in rabbit plasma.

| Concentration  (ng mL^-1^) | Recovery ± SD^*^ | % RSD |
| --- | --- | --- |
| 4.95 (LLOQ) | 96.52 ± 0.98 | 1.12 |
| 10 (LQC) | 98.4 ± 0.15 | 0.15 |
| 100 (MQC) | 98.3± 0.07 | 0.071 |
| 150 (HQC) | 98.8 ± 0.49 | 0.50 |

^*^ Average of three determinations.

**Table 2S.** The repeatability and reproducibility of the proposed MSDLLME based RP-HPLC method for the determination of DZ in rabbit plasma

| Concentration (ng mL^-1^) | Repeatability | | Reproducibility | |
| --- | --- | --- | --- | --- |
|  | %Recovery ± SD* | % RSD | %Recovery ± SD* | % RSD |
| 4.95 (LLOQ) | 96.12 ± 1.72 | 1.17 | 95.95 ± 1.92 | 1.24 |
| 10 (LQC) | 99.7 ± 0.94 | 0.94 | 98.9 ± 0.87 | 0.88 |
| 100 (MQC) | 100.1 ± 0.43 | 0.43 | 99.9 ± 0.72 | 0.72 |
| 150 (HQC) | 99.5 ± 0.57 | 0.57 | 98.9 ± 1.12 | 1.13 |

^*^ Average of six determinations.
